# Urban fragmentation leads to lower floral diversity, with knock-on impacts on bee biodiversity

**DOI:** 10.1038/s41598-020-78736-x

**Published:** 2020-12-10

**Authors:** Panagiotis Theodorou, Sarah-Christine Herbst, Belinda Kahnt, Patricia Landaverde-González, Lucie M. Baltz, Julia Osterman, Robert J. Paxton

**Affiliations:** 1 General Zoology, Institute for Biology, Martin-Luther University Halle-Wittenberg, Hoher Weg 8, 06120 Halle (Saale), Germany; 2 Unidad para el Conocimiento, Uso y Valoración de la Biodiversidad, Centro de Estudios Conservacionistas–CECON-, Facultad de Ciencias Químicas y Farmacia, Universidad de San Carlos de Guatemala, Avenida La Reforma 0-63 zona 10, 01010 Ciudad de Guatemala, Guatemala; 3 Helmholtz Centre for Environmental Research-UFZ Leipzig, ESCALATE, Department of Computational Landscape Ecology, Permoserstrasse 15, 04318 Leipzig, Germany; 4 German Centre for Integrative Biodiversity Research (iDiv) Halle-Jena-Leipzig, Deutscher Platz 5e, 04103 Leipzig, Germany

**Keywords:** Biodiversity, Community ecology, Urban ecology

## Abstract

Bees and flowering plants are two closely interacting groups of organisms. Habitat loss and fragmentation associated with urbanisation are major threats to both partners. Yet how and why bee and floral richness and diversity co-vary within the urban landscape remain unclear. Here, we sampled bees and flowering plants in urban green spaces to investigate how bee and flowering plant species richness, their phylogenetic diversity and pollination-relevant functional trait diversity influence each other in response to urban fragmentation. As expected, bee abundance and richness were positively related to flowering plant richness, with bee body size (but not bee richness and diversity) increasing with nectar-holder depth of flowering plants. Causal modelling indicated that bottom-up effects dictated patterns of bee-flower relationships, with urban fragmentation diminishing flowering plants richness and thereby indirectly reducing bee species richness and abundance. The close relationship between bees and flowering plants highlights the risks of their parallel declines in response to land-use change within the urban landscape.

## Introduction

Urbanisation, defined as the process by which people congregate in cities and, by extension, those cities grow and expand^1^, is one form of anthropogenic land-use alteration with overall negative effects on biodiversity^2^. Urban areas are one of the fastest growing ecosystems on Earth, with the development of cities influencing many aspects of the environment. Urbanisation leads to greater habitat fragmentation, degradation and loss, increased pollution and more impervious surfaces compared to non-urban habitats^3^. Such extreme land-use alteration can directly affect species persistence, alter competition and predation dynamics^4,5^ and influence species evolutionary change, including that of wild bees and native flowering plants^6–8^.

Pollinators, including bees, rely on flowers for food largely in the form of pollen and nectar whilst many flowering plants are dependent on pollinators for reproduction^9^. Previous studies have documented a strong correlation between flowering plant species richness in a community and pollinator species richness^10–14^. Biesmeijer et al.^15^ observed parallel declines in insect pollinators and insect-pollinated plants in Britain and the Netherlands, suggesting a causal relationship in which the extinction of one leads to extinction of the other, though the direction of causality was not made clear. Increases in flowering plant richness and abundance have been shown to enhance temporal stability in pollinator communities, thereby reducing extinction risk^13,16^. Similarly, studies have provided evidence that pollinator biodiversity can affect overall flowering plant diversity, density, reproduction, seedling diversity and abundance^17–20^.

Flowering plant and pollinator interactions form complex networks that are partly determined by the matching of phenotypic traits of the interacting species^21^. As a consequence, functional traits that regulate plant-pollinator interactions could control niche breadth and the partitioning of interaction partners for both the plants and the pollinators^22^. This could lead to interrelationships between functional diversity of the two partners through biotic filtering and competitive exclusion^22^. Indeed, Papanikolaou et al.^19^ and Albrecht et al.^22^ have previously reported a strong relationship between functional diversity of plant and pollinator communities. Moreover, if functional distances of the focal traits in the interacting partners are proportional to the time since species diverged, then another metric of community diversity, phylogenetic diversity, might also be expected to be correlated between the two interacting partners.

Land-use change, including urbanisation, can affect both native plant and bee species community structure through the biotic and abiotic factors affecting both interacting partners, and consequently their mutualistic network structure^23,24^. Studies have shown that urbanisation can have varying effects on native bee species richness: negative, neutral or even positive^14,25–28^. Urbanisation can also affect bee functional diversity^29–31^, benefiting parasitic and social, cavity-nesting, generalist and large-bodied bee species as well as exotic species^8,27,32–35^. Moreover, urbanisation can have mixed effects on native plant species richness^36,37^, with German cities being very rich with regard to native plant species, potentially because they are located in areas of high geological diversity^37^. Plant functional traits and phylogenetic diversity have also been shown to be affected by urbanisation^36,38,39^. Biennial, self- or wind-pollinated, plants with scleromorphic or succulent leaves, neophytes, cr-strategists (competitors/ruderals) and cs-strategists (competitors/stress tolerators) as well as exotic species are more frequent in urbanized than in non-urban areas and also more phylogenetically diverse in cities^36,39,40^. However, despite the growing body of research on flowering plants and bees in urban areas, little is known to date about how bee and floral diversity co-vary within the urban landscape.

In this study, we addressed this knowledge gap by quantifying local floral and bee diversity in urban sites to evaluate plant-bee diversity relationships and to examine how local habitat factors and the structure of urban land-use affect—directly or indirectly—plant and bee communities. We aimed to test for direct effects of urban habitat composition and fragmentation on both interacting partners and then to evaluate whether indirect effects are mediated by bee diversity influencing flowering plant diversity or by flowering plant diversity influencing bee diversity (i.e. the direction of causality in the plant-bee biodiversity relationship). We predicted that urban fragmentation would have negative effects on the abundance, species richness, functional and phylogenetic diversity of both bees and flowering plants. Due to the strong interrelationship between flowering plants and bees, we also predicted that plant-bee biodiversity would co-vary. Simulation models of plant and pollinator coextinctions have suggested that mutualistic networks are more robust to animal extinction than to plant extinction and revealed that plant communities are very resilient to pollinator losses^41^. This might be due to the often higher number of animal pollinator species compared to plant species in plant-pollinator networks and because pollination is characterised by high degree of animal redundancy^41–43^. In addition, the development of alternative reproduction strategies in plants (e.g. self-pollination, seed bank, clonal propagation), further argues that plants might depend less on their animal pollinators than vice versa. We therefore predicted a bottom-up (from plants to bees) instead of a top-down (from bees to plants) direction of causality in the plant-bee biodiversity relationship.

## Results

### Bee and flowering plant communities

We sampled local communities of flowering plants and bees monthly between June and August 2017 using transect sampling in eight urban semi-natural sites. From a total of 1440 min of transect sampling, we collected a total of 845 wild bee specimens representing 63 species from 23 genera within six families (Supplementary Table S1). The majority of these specimens (326, 39%) were bumblebees (*Bombus* spp.), 140 (17%) were from the genus *Halictus*, 108 (13%) were from the genus *Lasioglossum*, 106 (13%) were from the genus *Hylaeus*, 36 (4%) were from the genus *Megachile*, 26 (3%) were from the genus *Dasypoda*, 25 (3%) were from the genus *Panurgus*, 22 (3%) were from the genus *Andrena*, and 20 (2%) were from the genus *Colletes*. The number of bee species sampled per sampling site and per transect walk ranged from 6 to 14 (mean ± SD = 9.91 ± 2.60). Additionally, from our transect sampling we collected 291 honey bee individuals. Due to the potential effect of honey bees on wild bee communities^44^, the abundance of honey bees per site was used as a predictor in all downstream analyses. Honey bee abundance was not, however, an important predictor in any of our statistical models.

The majority of bee species sampled were ground nesters (45 out of 63 species, 71.42%; 553 out of 845 individuals, 65.44%) with a small body size (intertegular distance; ITD less than 2600 μm; 42 out of 63 species, 66.66%; 445 out of 845 individuals, 52.66%). Most bee species were solitary (41 out of 63 species, 65.07%; 390 out of 845 individuals, 46.15%), followed by social (16 species, 25.39%; 443 out of 845 individuals, 52.42%) and parasitic species (6 species, 9.52%; 12 out of 845 individuals, 1.42%). For the remaining functional categories, we found more polylectic (40 out of 63 species, 63.49%; 653 out of 845 individuals, 77.27%) than oligolectic species (17 out of 63 species, 26.98%; 180 out of 845 individuals, 21.30%) and more short-tongued (34 out of 63 species, 53.96%; 450 out of 845 individuals, 53.25%) than long-tongued species (29 out of 63 species, 46.03%; 395 out of 845 individuals, 46.74%).

We sampled a total of 58 flowering plant species in flower (Supplementary Table S2). The most widespread were *Hypericum perforatum*, *Daucus carota*, *Rubus fruticosus*, *Hieracium lachenalii* and *Tanacetum vulgare*. The number of plant species in flower per sampling site and per transect walk ranged from 5 to 17 (mean ± SD = 10.92 ± 3.19). Most had an open flower shape (36, 62.06%), followed by plants with papilionaceous (19, 32.75%) and tubular flower shape (3, 5.17%). The majority of the plants sampled had a yellow flower colour (22, 37.93%), followed by violet (17, 29.31%), white (16, 27.58%) and red (3, 5.17%). Furthermore, most of the plants were perennials (38, 65.51%) and allogamous (38, 65.51%) with protandrous (30, 51.72%) or homogamous (22, 37.93%) flower sex timing.

### Local (patch) and landscape scale effects on bee communities

**Figure 1:**
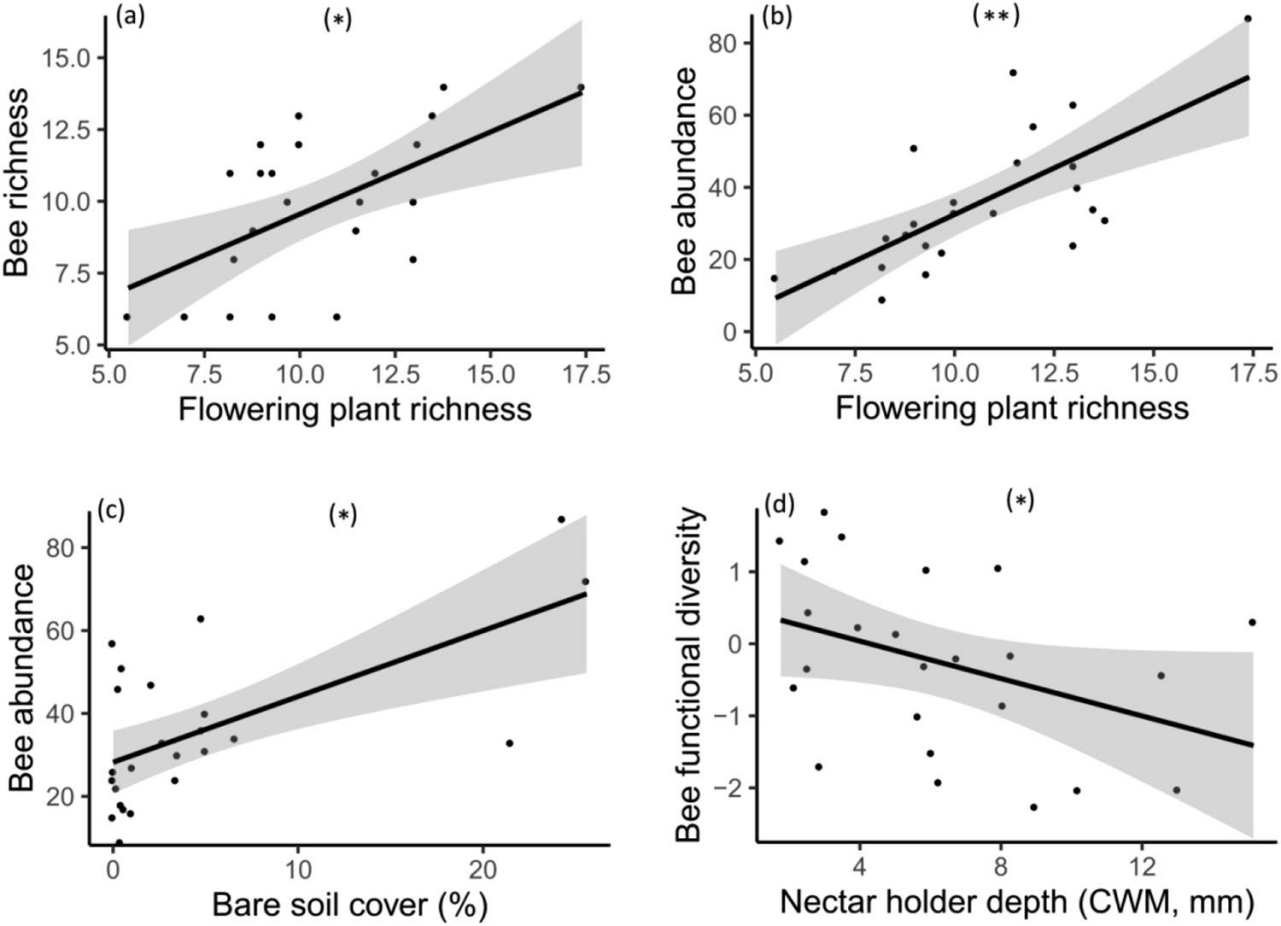
Relationships between (**a**) bee species richness and flowering plant species richness, (**b**) bee abundance and flowering plant species richness, (**c**) bee abundance and bare soil cover, (**d**) bee functional diversity and nectar holder depth. Plotted lines show the predicted relationship and shaded areas indicate the 95% confidence intervals: *P ≤ 0.05; **P ≤ 0.01.

**Figure 2:**
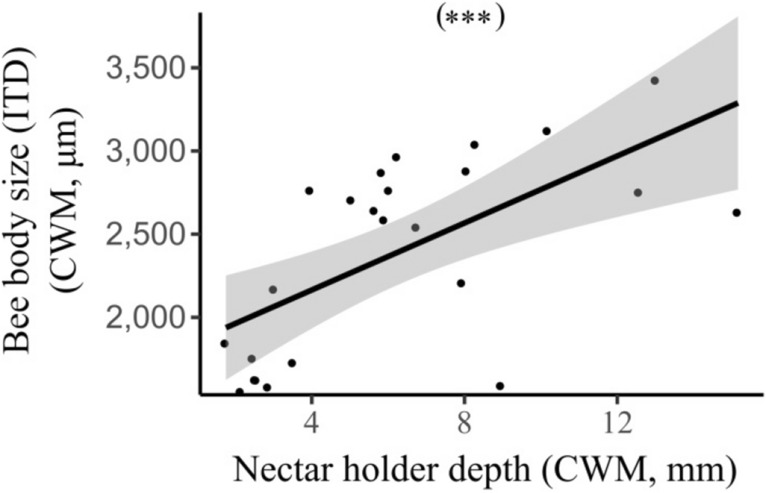
Relationship between community weighted mean of bee intertegular distance (ITD) and community weighted mean (CWM) of flowering plant nectar holder depth. Plotted lines show the predicted relationship and shaded areas indicate the 95% confidence intervals: ***P ≤ 0.001.

We calculated flowering plant and bee diversity metrics (i.e. abundance, richness, functional diversity and phylogenetic diversity) and quantified local and landscape environmental variables (i.e. bare soil, landscape composition and fragmentation). We first explored the main flowering plant community diversity metrics and environmental drivers of bee diversity. We found a variety of relationships between bee communities and flowering plant diversity, bare soil cover and landscape heterogeneity. Firstly, bee species richness was positively related to flowering plant richness (Table 1; Fig. 1a). Overall bee abundance was also positively associated with flowering plant richness and the percentage of bare soil cover (Table 1; Fig. 1b,c). Bee functional diversity decreased with increasing community weighted mean (CWM) nectar holder depth (Table 1; Fig. 1d). The CWM of bee intertegular distance (ITD) was, though, positively related to the CWM of plant nectar holder depth (Table 1; Fig. 2) as well as local flower richness (Table 1). Therefore diverse flowers equated with diverse bees. Yet as mean corolla length increased, bee communities became less diverse and with a larger average body size. We did not find any important predictor for bee phylogenetic diversity (Table 1). Landscape composition, edge density, habitat fragmentation, overall plant phylogenetic and functional trait diversity were not included in the most parsimonious models of overall bee abundance and richness, bee functional and phylogenetic diversity.

**Table 1:** Model selection statistics and model averaging coefficients (full average) for flowering plant and bee biodiversity metrics.

Response	Best model	AICc	ΔAICc	Weight	Factor	Beta coefficient	*P* value
**Bees**							
(a) Species richness	⁓ Plant richness + Bee abundance	121.69	0.00	0.17	Plant richness	0.148	0.021*
					Bee abundance	0.110	0.030*
(b) Abundance	⁓ % Bare soil + Plant richness	197.19	0.00	0.23	% Bare soil	0.186	0.017*
					Plant richness	0.266	0.001**
(c) Functional diversity	⁓ Plant CWM of nectar holder depth	84.18	0.00	0.13	Plant CWM of nectar holder depth	− 0.462	0.023*
(d) CWM of body size	⁓ Plant richness + Plant CWM of nectar holder depth	326.53	0.00	0.29	Plant richness	236.06	0.026*
	⁓ Plant richness + Plant CWM of nectar holder depth + Plant phylogenetic diversity	327.75	1.22	0.16	Plant CWM of nectar holder depth	393.85	< 0.001***
	⁓ Plant richness + Plant CWM of nectar holder depth + Plant functional diversity	328.10	1.58	0.13	Plant phylogenetic diversity	− 165.80	0.105^ns^
					Plant functional diversity	169.80	0.115^ns^
(e) Phylogenetic diversity	⁓ Intercept-Only	59.90	0.00	0.28	–	–	–
**Flowering plants**							
(f) Species richness	⁓ Bee abundance + Fragmentation	106.60	0.00	0.15	Bee abundance	1.611	< 0.001***
					Fragmentation	− 0.866	0.019*
(g) Functional diversity	⁓ Fragmentation	86.52	0.00	0.22	Fragmentation	− 1.076	< 0.001***
(h) Phylogenetic diversity	⁓ Fragmentation	73.30	0.00	0.31	Fragmentation	− 0.698	0.002**
(i) CWM of nectar holder depth	⁓ Bee CWM of body size	123.71	0.00	0.24	Bee CWM of body size	2.136	< 0.001***
	⁓ Bee CWM of body size + Fragmentation	125.45	1.75	0.10	Fragmentation	− 0.203	0.693^ns^

ns = not significant; *P ≤ 0.05; **P ≤ 0.01; ***P ≤ 0.001.

**Figure 3:**
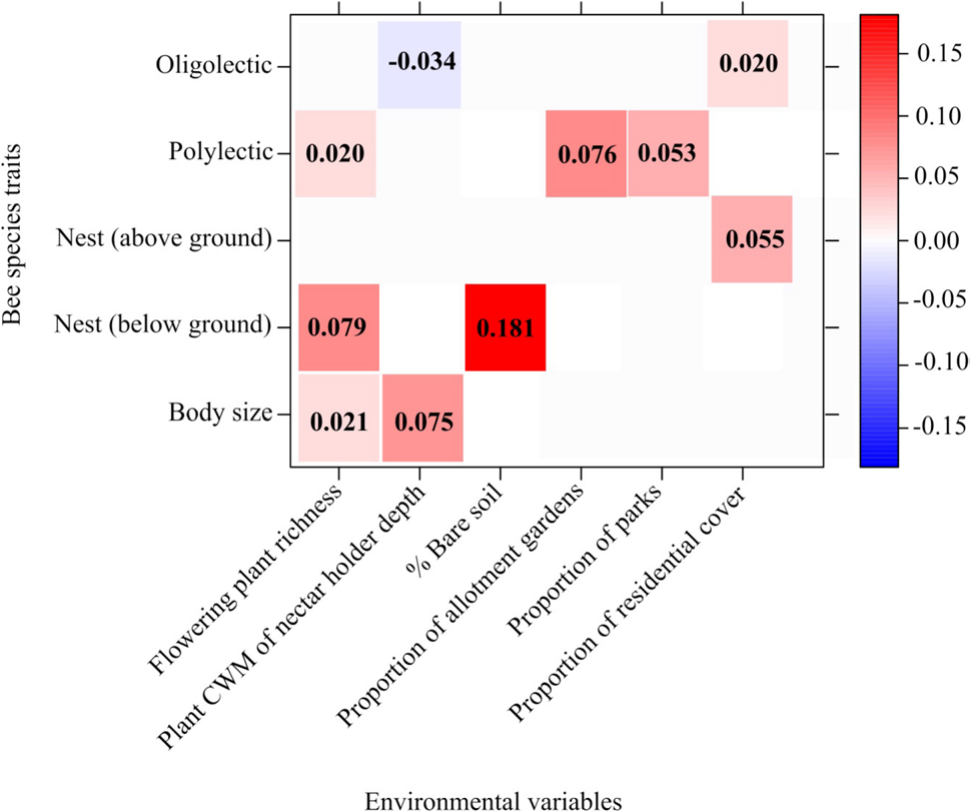
Relationships between bee traits and environmental variables. Red cells indicate positive relationships and blue cells indicate negative relationships. Colour depth indicates the strength of the trait-variable association. Empty cells indicate no relationship. The numbers within red and blue cells correspond to regression coefficients.

As bee species responses to the availability and distribution of resources could be trait-specific, we used fourth-corner analysis and explored the relationships between the abundance of each functional group and local flowering plant diversity, bare soil cover and landscape scale factors. Due to the strong correlation among bee traits (i.e. polylectic bees were mostly social, long-tongued bees were usually large, and above-ground nesters were mostly solitary and with a long tongue (Supplementary Table S3), we ran fourth-corner analysis only for lecty, nesting and body size. Fourth-corner analysis revealed numerous associations between the abundance of different bee functional groups, floral and environmental variables (model deviance = 48.03, *P* = 0.004; Fig. 3). Oligolectic bee species abundance was positively related to the proportion of residential cover, including domestic gardens (Fig. 3). In contrast, oligolectic bee species abundance was negatively related to the CWM of nectar holder depth (Fig. 3). Polylectic bee species abundance was positively associated with local flowering plant richness, the proportion of allotment gardens, and the proportion of parks (Fig. 3). Above ground nesting bee abundance was positively associated with the proportion of residential cover (domestic housing with gardens) (Fig. 3). Ground nesting bee abundance was positively associated with local flowering plant richness and the percentage of bare soil cover (Fig. 3). Bee body size (ITD) was also positively associated with local flowering plant richness and the CWM of nectar holder depth (Fig. 3).

### Bee and landscape scale effects on flowering plant communities

**Figure 4:**
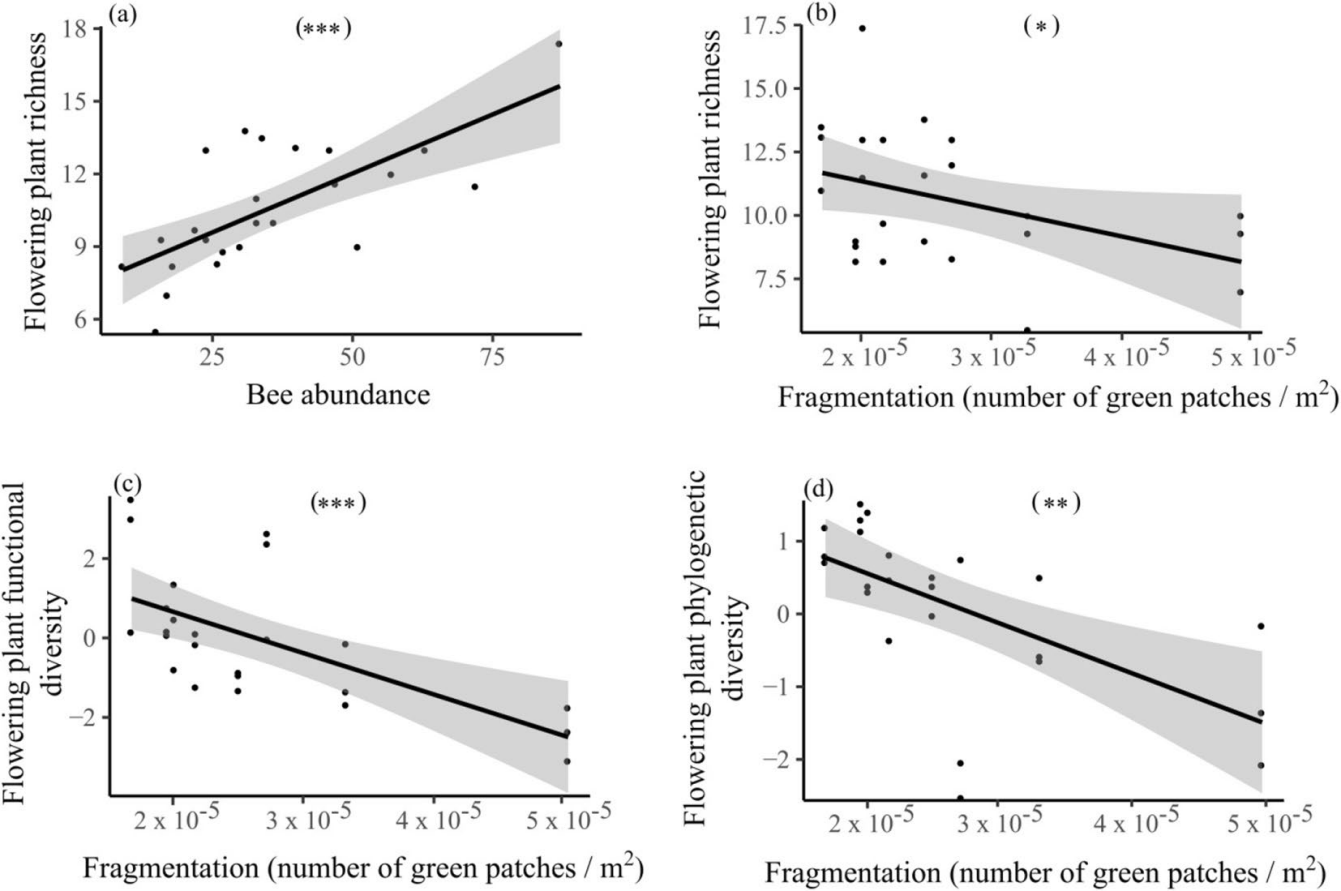
Relationships between (**a**) flowering plant species richness and bee abundance, (**b**) flowering plant species richness and fragmentation (number of disconnected green patches divided by their total surface area), (**c**) flowering plant functional diversity and fragmentation and (**d**) flowering plant phylogenetic diversity and fragmentation. Plotted lines show the predicted relationship and shaded areas indicate the 95% confidence intervals: *P ≤ 0.05; **P ≤ 0.01; ***P ≤ 0.001.

We also explored the main bee community diversity metrics and environmental drivers of plant communities. Plant species richness was positively related to bee abundance and negatively related to landscape fragmentation (Table 1; Fig. 4a,b). Flowering plant functional diversity was also lower in more fragmented landscapes (Table 1; Fig. 4c). Plant phylogenetic diversity was also negatively related to urban landscape fragmentation (Table 1; Fig. 4d). As expected, the community level weighted mean of nectar holder depth was positively associated with the CWM of bee intertegular distance (Table 1). Landscape composition, edge density, overall bee phylogenetic and functional trait diversity were not included in the most parsimonious models of flowering plant richness, functional and phylogenetic diversity.

### Structural equation modeling

**Figure 5:**
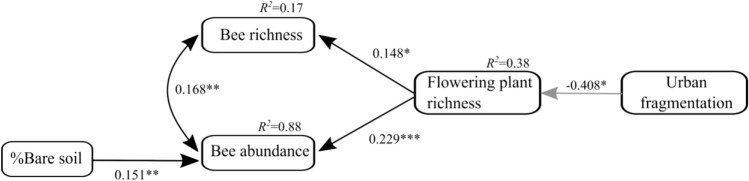
Final path model of local (patch) factors, landscape heterogeneity and their relationships with bee richness and abundance. Black solid arrows show positive and grey arrows negative direct effects as derived from the piecewise SEM analysis. Standardized path coefficients are reported next to the bold arrows and *R*^2^ values (percentage of explained variation) are reported for all response variables. *P ≤ 0.05; **P ≤ 0.01; ***P ≤ 0.001.

To reveal those putative causal factors, or interactions among them, that influenced plant and bee communities and to infer causality in plant-bee relationships within the urban ecosystem, we used structural equation modeling (SEM). Our piecewise SEM method confirmed the strong relationships between bee biodiversity, local ground nesting resources, floral richness and urban fragmentation. In the best version of the SEM, with a stable fit to our data (AIC_plants→bees_ = 33.387, Fisher’s C = 9.387, d.f. = 8, *P* = 0.311; Fig. 5), flowering plant richness had a positive impact on overall bee richness (*P* = 0.02; Fig. 5). In addition, bee abundance was driven by both flowering plant richness and bare soil cover (*P* < 0.01; Fig. 5). Flowering plant richness itself was negatively impacted by urban habitat fragmentation (*P* = 0.02, Fig. 5). The relationship between bee richness and abundance and flowering plant richness resulted in an indirect negative effect of habitat fragmentation on bees (Sobel test; − 0.06, *P* = 0.04 for bee richness and − 0.09, *P* = 0.02 for bee abundance) mediated by its impact on flowering plant richness. The alternative SEM with the direction of relationship from bees to flowering plants had a higher AIC and considerably reduced support (AIC_bees→plants_ = 38.328 versus AIC_plants→bees_ = 33.387; AIC_bees→plants_ − AIC_plants→bees_ = 4.941).

## Discussion

Our sampling in urban semi-natural sites revealed strong relationships between flowering plant and bee diversity. We furthermore found urban fragmentation to be negatively related to flowering plant species richness, functional and phylogenetic diversity. In accordance to our expectations, our causal modeling identified bottom-up effects (from flowering plants to bees) to dominate the relationship between flowering plant and bee diversity. Most importantly, our causal modeling revealed an indirect negative effect of urban fragmentation on bee diversity that was mediated via its negative effects on flowering plant richness. We expand on these results and discuss their ecological implications.

We found marked relationships between overall bee richness and local flowering plant richness and between overall bee abundance, flowering plant richness and bare soil cover. This reflects the strong dependence of bees on the local availability not only of food (the richness of flowering plants) but also of nesting resources, namely the availability of bare soil cover for below-ground nesting bees, as has been previously reported for semi-natural and urban ecosystems^10,11,13,14,32,45–47^. Additionally, our results are in line with previous studies suggesting that bees might respond positively to local floral and nesting resources, irrespective of surrounding land-use change^14^.

Resource availability is a strong determinant of community assembly, and bee species may respond differently depending on how resources are distributed at different scales^48^. Investigating patterns of bee biodiversity across broad scales could potentially mask trends in bee functional guilds at smaller scales, knowledge of which could be important for bee conservation actions within the urban environment. Bees are “central place foragers” (i.e. females carry resources back to their nest); as such, their resource needs must be met within their flight range limits. Large-bodied species with large foraging ranges might respond to the resource distribution not only at small but also at larger scales. In contrast, small-bodied species with short home ranges might only be able to respond to resource availability locally. Furthermore, species that depend on two or more resources (e.g. nesting and food resources) that vary in their distribution patterns might respond to each resource at a different scale. In addition to the availability of local floral and nesting resources, our data also showed that many of our sampled species also responded to the availability of resources at larger scales in a trait-dependent manner. Several of the surrounding urban green land-uses had a positive effect on the abundance of many of the bee functional groups sampled. Our results suggest that parks, allotment gardens and residential areas can all be important habitats for bees^49^ and point to the importance of the availability of resources (i.e. food and nesting) at both the local and the landscape scales for bee communities within the urban ecosystem.

We found that bee responses to urbanisation were dependent on species life history traits, especially those related to foraging and nesting preferences. Previous studies have documented that urban areas benefit cavity-(above ground) nesting and generalist bee species^26,27,32–34^. Yet, in our study we found a greater abundance of ground nesting bees in urban areas. This suggests that not only urban residential, public and private gardens^33,49,50^ but that low management fragments of semi-natural vegetation could also be important habitats within the urban ecosystem which benefit ground nesting bees. Furthermore, we found more polylectic compared to oligolectic bee species. Although flowering plant richness could be high in urban areas^37^, it seems that it may only benefit generalist foragers^14^.

Urban landscape structure did not appear to relate to bee functional or phylogenetic diversity. Indeed, the CWM of flowering plant nectar holder depth was the only significant predictor of bee functional diversity, which decreased as the CWM of flowering plant nectar holder depth increased. Functional traits related to size are important predictors of mutualistic networks and can act as barriers preventing interactions^22,51^. The increase in the CWM nectar holder depth seems to act as a major filter on the wild bee community, leading to trait convergence favouring long-tongued, large and social bee species, traits that we also found to be strongly correlated among bee species. The lack of a relationship between overall flowering plant functional diversity and bee functional diversity and the strong relationship between the CWM of bee intertegular distance and the CWM of flowering plant nectar holder depth suggest that the majority of the flowering plant traits analysed were unimportant for structuring bee communities. They suggest that size constraints have the strongest effect on bee and floral community assembly.

During urbanisation, semi-natural areas are converted into those with impervious surfaces, resulting in habitat loss^3^. Furthermore, the development of roads, railways and impervious surfaces results in habitat fragmentation^52^. Urban habitat loss and fragmentation reduce the size and increase the isolation of plant populations, which makes species more vulnerable to extinction^53^. In contrast to the bees, we found a strong negative and direct impact of urban fragmentation on flowering plant richness. Moreover, fragmentation resulted in flowering plant functional convergence and phylogenetic clustering.

Although we did not find direct effects of fragmentation on bee biodiversity, our findings suggest plant-mediated effects of urban fragmentation on bees. Bees are well adapted to shift between patchy foraging and nesting resources and often benefit from habitat edges between patch types^28,54^. Nonetheless, depletion and extensive fragmentation of floral food resources at local and landscape scales could lead to local bee population extinctions and limit recolonizations, with negative effects on bee abundance and richness^55^. Accordingly, our results revealed more pronounced effects of flowering plants on bees than vice versa. This suggests that flowering plants have a reduced dependency on pollinators than pollinators do on plants, potentially due to the alternative reproduction strategies of many plants such as clonal propagation, self-fertilisation and seed banking. Our results point to the importance of considering indirect effects mediated by species interactions when assessing species’ responses to land-use changes, including that of urbanisation, and further highlight the risks of parallel declines between wild bees and flowering plants. From an applied perspective, our results have important implications for pollinator conservation in cities. The strong effects of flower resource richness on bee communities suggest that simple local management practices to increase the diversity of native plants could benefit wild bees in urbanised areas.

## Materials and methods

### Study sites

Our study was conducted in the city of Halle (Saale), with a population of approximately 240,000 inhabitants. It covers an area of about 13,000 hectares, of which most (37.7%) is paved, (buildings and open areas: 3446 ha, streets: 1646 ha), 25.6% is adjacent agricultural land (3455 ha), 16.9% consists of forest (2292 ha) and 14.1% of parks (1904 ha).

Using land-cover maps obtained from Geofabrik GmbH (http://www.geofabrik.de/) and Quantum GIS^56^, we pre-selected eight independent sites with semi-natural vegetation that differed in their surrounding proportion of urban cover (i.e. residential cover and amount of impervious surfaces) (Supplementary Fig. 1; Supplementary Table S4). All sites were of low management, without obvious differences in shading or sources of pollution. No mowing was performed prior to or during our sampling in any of the sites. We ensured a minimum distance of 1.5 km between sites, sufficient for them to be considered independent^57^.

### Sampling flowering plant and bee diversity

Local communities of flowering plants and bees were sampled between 10th June and 18th August 2017 using 30 min transect walks at each of the eight sites. In every month (June, July and August), we conducted two rounds of sampling in each site, one round in the morning between 9:00 and 12:00 and one round in the afternoon between 13:00 and 16:00. Hence, every site was visited a total of six times. For the transect sampling we used the variable transect walk method, as described by Westphal et al*.*^58^. In each of the urban semi-natural sites, the collector was not restricted to a fixed transect line; instead they walked for 30 min at a slow steady pace among any potential floral resource patch and collected bees visiting flowers as well as the flower that was visited. In comparison to fixed transect walks, this method overcomes potential undersampling due to the spatiotemporal variation in bees and floral resources^58^. Furthermore, in a comparative study across geographic regions in Europe, variable transects walks were found to be more efficient with respect to sample coverage, bee species richness and abundance in comparison to fixed transect walks. We performed our transect walks only on sunny days with clear skies, wind speed less than 7 m/s and a temperature above 17 °C. Sampling was performed by the same collector. Bees were collected in tubes with 96% ethanol and stored at − 20 °C for later identification. We collected whole botanical specimens, including leaves and reproductive structures of the flowering plants. We identified bees to species using identification keys^59–61^ and DNA barcoding of the COI gene^62^ (Supplementary Table S1; Supplementary Methods 1). All flowering plants were identified to species using identification keys for the local flora^63,64^ (Supplementary Table S2). Bee specimens are kept at the General Zoology research group, Martin-Luther University Halle-Wittenberg. Sequences obtained from COI barcoding were submitted to the NCBI GenBank database and are available under Accession Numbers MW065567–MW065776.

### Flowering plant and bee diversity metrics

We calculated four metrics of community diversity: abundance, species richness, functional diversity and phylogenetic diversity. Abundance was calculated as the total number of every bee individual sampled in each site. Bee and flowering plant species richness were calculated as the number of species of each taxon in each site. To estimate functional diversity, for every individual bee sampled we quantified body size as intertegular distance (ITD) to the nearest 10 μm using an OLYMPUS SZX7 stereo-microscope and the software cellSens standard v.1.6 (Tokyo, Japan). In addition, using the available literature^65^, we obtained five bee functional traits for each species (sociality, tongue length, nesting behaviour, voltinism and lecty; Table 2).

**Table 2:** Description of (a) bee functional traits and (b) flowering plant functional traits used in this study.

Traits	Description
**(a) Bees**	
Body size	Intertegular distance (µm)
Sociality	Three categories: Solitary, social, parasitic
Tongue length	Two categories: Long (> 3 mm), short (< 3 mm)
Nesting behaviour	Two categories: Above ground nesting (cavity, stem, wood), below ground nesting (within existing tunnels or excavators)
Voltinism	Two categories: Univoltine, bivoltine
Lecty	Two categories: Oligolectic, polylectic
**(b) Flowering plants**	
Nectar holder depth	Quantitative (mm)
Flower colour	Four categories: Blue–violet, red–pink, white, yellow–orange
Flower shape	Three categories: Open, tubular, papilionaceous
Breeding system	Three categories: Allogamous, autogamous, mixed mating
Flower sex timing	Three categories: Protandrous, protogynous, homogamous
Plant longevity	Four categories: Annual, annual/biennial, biennial, perennial

For every flower collected, we measured the nectar holder depth (the maximal depth between the base of flower, where nectar is stored, and where the flower opens and is accessible to all insects) using a Vernier calliper to the nearest 10 μm. Additionally, we used two qualitative measures to characterise every flower: (1) flower restrictiveness (the accessibility of a flower for a plant-flower visitor due to its shape) and (2) flower colour. Flower restrictiveness was classified into three categories: (i) open or bowl-shaped flowers, (ii) bell shaped, short tubular or long narrow tubular flowers, and (iii) papilionaceous flowers. Using the TRY database v. 4.1^66^ (see Supplementary Table S2 for detailed references) we extracted three additional pollination-related plant functional traits: breeding system, flower sex timing and plant longevity (Table 2).

As a measure of functional diversity we used Rao’s quadratic entropy (Rao’s Q)^67,68^, calculated using the R package *FD*^68^. Community weighted mean (CWM) trait values for intertegular distance and nectar holder depth were also calculated using the R package *FD*. As functional diversity is partly confounded by species richness, we used a null model approach to obtain an unbiased standardised functional diversity estimate per site^69^. For a given number of species, this approach creates a random (null) distribution of functional diversity values. Holding species richness constant for each site, it uses randomly selected species from the entire species pool to calculate a null functional diversity for each richness level. We repeated the procedure 1000 times to produce a distribution of null values. The standardised Rao’s Q was calculated for each site as; (*N − N̅*_*r*_)/*σ*_*Nr*_, where *N* is the observed value of Rao’s Q and *N̅*_*r*_ and *σ*_*Nr*_ are the mean and standard deviation, respectively, for the 1000 replicated randomised (null) Rao’s Q values. Standardised Rao’s Q values > 0 suggest that traits are more divergent than expected by chance whereas Rao’s Q values < 0 suggest traits are more convergent than expected by chance. Finally, if Rao’s Q values ≈ 0, then trait values are not different from random expectations.

To estimate phylogenetic diversity we build bee and plant phylogenies based on two genes for each taxon (bee: COI and elongation factor 1-alpha [ef1-alpha]; plant: ribulose-1,5-bisphosphate carboxylase/oxygenase large subunit [rbcL] and maturase K [matK]), which were downloaded from NCBI GenBank (see Supplementary Methods 2). To estimate the total evolutionary history shared across all species within a community (i.e. phylogenetic diversity), we used Faith’s phylogenetic diversity index (PD;^70^). PD is the phylogenetic analogue of taxon richness and is defined as the minimum total length of all the phylogenetic branches required to span a given set of taxa on the phylogenetic tree^70^. Similarly to functional diversity, PD and species richness are strongly correlated and we thus used the same null model approach used for functional diversity to obtain standardize PD measurements. The standardized PD was calculated by dividing the difference between the observed and expected PD by the standard deviation of the null distribution. Standardised PD values > 0 indicate phylogenetic dispersion and values < 0 indicate phylogenetic clustering.

### Local (patch) and landscape variables

To quantify local (patch) environmental variables, we randomly placed ten 1 m^2^ quadrats in every site and on every sampling visit. Within each quadrat, we measured the percentage of exposed soil, quantified as a surrogate for the availability of nesting opportunities for ground nesting bees like Andrenidae, Colletidae and Halictidae^45^. Additionally, we quantified patch size (i.e. the size of the continuous area of green or semi-natural vegetation at our sampling site) for every site using Google Earth Pro v.7.3.0 (Supplementary Table S4).

At each sampling site we estimated habitat heterogeneity at six spatial scales (250, 500, 750, 1000, 1250, and 1500 m) using Quantum GIS^56^ and land cover data obtained from Geofabrik GmbH (http://www.geofabrik.de/). The accuracy of the data was confirmed using onsite surveys and by matching the shapefile’s features with Google earth images. We calculated landscape diversity (*H*_*s*_) for each site at each radius as: *H*_*s*_ = − ∑*p*_*i*_ × ln *p*_*i*_, where *p*_*i*_ is the proportion of each land cover of type *i*.

We quantified landscape heterogeneity with a number of metrics known to impact flowering plant-bee interactions^14,49^. These metrics were: (i) the proportion of parks (ii) the proportion of forest cover, (iii) the proportion of allotment gardens (iv) the proportion of semi-natural areas (meadows, vacant lots, remnants), (v) the extent of agricultural cover, (vi) the proportion of residential cover (domestic housing with gardens), (vii) edge density, as the total length of green patch edges divided by the total area, which quantifies landscape configuration, and (viii) number of disconnected green patches divided by their total surface area as an indicator of the degree of fragmentation.

To detect the spatial scale at which land cover had the most power to explain flowering plant and bee occurrence, we correlated flowering plant and bee diversity with landscape Shannon diversity in each of our eight sites at all six spatial scales and compared the resulting correlation coefficients. Correlation coefficients peaked at 1000 m radius for both interacting partners (Supplementary Table S5), which was then chosen as the spatial scale for subsequent statistical modelling. This scale is within the foraging range of many bee species^57^.

### Statistical analyses

We tested for local (patch) and landscape scale effects on each bee and plant diversity metrics using linear mixed effects models (LMMs) and generalised linear mixed models (GLMMs). We used LMMs for functional diversity and phylogenetic diversity and GLMMs with a Poisson error structure for abundance and richness. Plant biodiversity metrics (i.e. richness, functional and phylogenetic diversity) were included as predictors to the models fitted to bee metrics and vice versa. Bee abundance was included as a covariate in the models for bee and plant species richness to control for sample size effects. In all models, month and site were included as random effect factors. We used an automated model selection approach (all subsets) based on the second-order Akaike Information Criterion corrected for small sample size (AICc), and allowing only up to three variables to avoid over-fitting, to identify relevant predictors for each biodiversity metric using the ‘dredge’ function in the R package *MuMIn*^71^. We used a cut-off ΔΑΙCc value of 2^72^ and, if more than one model was retained, we used model averaging (function ‘model.avg’;^71^). All mixed model analyses were performed using the package *lme4*^73^.

To explore relationships between bee functional traits and environmental variables (flowering plant diversity metrics, percentage of exposed soil and landscape heterogeneity) we used fourth-corner analysis^74^ using the ‘traitglm’ function within the R package *mvabund*^75^. Multivariate generalised linear fourth-corner models were fitted with a negative binomial distribution and a least absolute shrinkage and selection operator’s (LASSO) penalty (i.e. method = glm1path). LASSO is an approach which penalises coefficients that do not reduce Bayesian Information Criteria (BIC) to zero. Analysis of model deviance was estimated using a Monte-Carlo resampling procedure (1000 resamples) to evaluate the global significance of trait-environment relationships.

In addition to our multiple regressions models, we performed piecewise structural equation modelling (SEM) in an attempt to infer causality in plant-bee biodiversity relationships. We hypothesised that local and landscape composition and configuration might have affected bee and flowering plant biodiversity directly and also indirectly through affecting each trophic level. We performed the piecewise SEM analysis using the R package *piecewiseSEM*^76^. Based on a priori knowledge of interactions and our multi-level modelling, we used the d-separation (d-sep) test to evaluate whether the non-hypothesised independent paths were significant and whether the models could be improved with the inclusion of any of the missing path(s). We used Fisher’s C statistic for evaluating the fit of piecewise SEM^77^. We constructed alternative structural equation models, changing the direction of causality from bees to flowering plants to that of flowering plants to bees. Models were compared using the Akaike information criterion (AIC) and Δ_*i*_. Δ_*i*_ is the difference between the AIC value of a given model against the value of the model with the lowest AIC_min_ (Δ_*i*_ = AIC_*i*_ − AIC_min_). Models with a Δ_*i*_ > 4 have little support^72^. Path coefficients and deviance explained were then calculated for the best structural equation model. We used Sobel’s method to test for significant indirect effects^78^.

Prior to all analyses we standardized all quantitative predictors. This was done to minimise potential effects of collinearity and to derive comparable estimates. Additionally we used variance inflation factors (VIFs) to check for collinearity among our explanatory variables. VIF was lower than 3 for all predictors, indicating no major effects of collinearity^79^. The residuals of all models were tested for spatial autocorrelation using Moran’s I, implemented in the R package *ape*^80^. Residuals were not found to be autocorrelated (*P* > 0.05 for all models). All statistical analyses were performed in R v. 3.5.2^81^. All model (GLMMs and LMMs) assumptions were checked visually and were found to conform to expectations (e.g. normality of the distribution of residuals, homogeneity of variances, linearity, non-overdispersion).

## Supplementary Information

Supplementary Information.

## Data Availability

All data are included as supplementary material.

## References

[1] Tisdale H (1942). The process of urbanization. Soc. Forces.

[2] McKinney ML (2002). Urbanization, biodiversity, and conservation. Bioscience.

[3] Grimm NB (2008). Global change and the ecology of cities. Science.

[4] Johnson MTJ, Munshi-South J (2017). Evolution of life in urban environments. Science.

[5] Turrini T, Sanders D, Knop E (2016). Effects of urbanization on direct and indirect interactions in a tri-trophic system. Ecol. Appl..

[6] Theodorou P (2018). Genome-wide single nucleotide polymorphism scan suggests adaptation to urbanization in an important pollinator, the red-tailed bumblebee (*Bombus lapidarius* L.). Proc. R. Soc. B Biol. Sci..

[7] Thompson KA, Renaudin M, Johnson MTJ (2016). Urbanization drives the evolution of parallel clines in plant populations. Proc. R. Soc. B Biol. Sci..

[8] Theodorou P, Baltz LM, Paxton RJ, Soro A (2020). Urbanisation is associated with shifts in bumblebee body size, with cascading effects on pollination. Evol. Appl..

[9] Ollerton J, Winfree R, Tarrant S (2011). How many flowering plants are pollinated by animals?. Oikos.

[10] Potts SG, Vulliamy B, Dafni A, Nee’man G, Willmer P (2003). Linking bees and flowers: how do floral communities structure pollinator communities?. Ecology.

[11] Steffan-Dewenter I, Tscharntke T (2001). Succession of bee communities on fallows. Ecography.

[12] Fründ J, Linsenmair KE, Blüthgen N (2010). Pollinator diversity and specialization in relation to flower diversity. Oikos.

[13] Ebeling A, Klein AM, Schumacher J, Weisser WW, Tscharntke T (2008). How does plant richness affect pollinator richness and temporal stability of flower visits?. Oikos.

[14] Theodorou P (2017). The structure of flower visitor networks in relation to pollination across an agricultural to urban gradient. Funct. Ecol..

[15] Biesmeijer JC (2006). Parallel declines in pollinators and insect-pollinated plants in Britain and the Netherlands. Science.

[16] Ghazoul J (2006). Floral diversity and the facilitation of pollination. J. Ecol..

[17] Clough Y (2014). Density of insect-pollinated grassland plants decreases with increasing surrounding land-use intensity. Ecol. Lett..

[18] Lundgren R, Totland Ø, Lázaro A (2016). Experimental simulation of pollinator decline causes community-wide reductions in seedling diversity and abundance. Ecology.

[19] Papanikolaou AD (2017). Wild bee and floral diversity co-vary in response to the direct and indirect impacts of land use. Ecosphere.

[20] Brosi BJ, Briggs HM (2013). Single pollinator species losses reduce floral fidelity and plant reproductive function. Proc. Natl. Acad. Sci. U. S. A..

[21] Vázquez DP, Blüthgen N, Cagnolo L, Chacoff NP (2009). Uniting pattern and process in plant–animal mutualistic networks: a review. Ann. Bot..

[22] Albrecht J (2018). Plant and animal functional diversity drive mutualistic network assembly across an elevational gradient. Nat. Commun..

[23] Kremen C (2007). Pollination and other ecosystem services produced by mobile organisms: a conceptual framework for the effects of land-use change. Ecol. Lett..

[24] Harrison T, Winfree R (2015). Urban drivers of plant-pollinator interactions. Funct. Ecol..

[25] Baldock KCR (2015). Where is the UK’s pollinator biodiversity? The importance of urban areas for flower-visiting insects. Proc. R. Soc. B Biol. Sci..

[26] Bates AJ (2011). Changing bee and hoverfly pollinator assemblages along an urban–rural gradient. PLoS ONE.

[27] Fortel L (2014). Decreasing abundance, increasing diversity and changing structure of the wild bee community (Hymenoptera: Anthophila) along an urbanization gradient. PLoS ONE.

[28] Theodorou P (2020). Urban areas as hotspots for bees and pollination but not a panacea for all insects. Nat. Commun..

[29] Buchholz S, Gathof AK, Grossmann AJ, Kowarik I, Fischer LK (2020). Wild bees in urban grasslands: urbanisation, functional diversity and species traits. Landsc. Urban Plan..

[30] Hung KJ, Ascher JS, Davids JA, Holway DA (2019). Ecological filtering in scrub fragments restructures the taxonomic and functional composition of native bee assemblages. Ecology.

[31] Buchholz S, Egerer MH (2020). Functional ecology of wild bees in cities: towards a better understanding of trait-urbanization relationships. Biodivers. Conserv..

[32] Cane JH, Minckley RL, Kervin LJ, Roulston TH, Williams NM (2006). Complex responses within a desert bee guild (Hymenoptera: Apiformes) to urban habitat fragmentation. Ecol. Appl..

[33] Banaszak-Cibicka W, Żmihorski M (2011). Wild bees along an urban gradient: winners and losers. J. Insect Conserv..

[34] Neame LA, Griswold T, Elle E (2013). Pollinator nesting guilds respond differently to urban habitat fragmentation in an oak-savannah ecosystem. Insect Conserv. Divers..

[35] Fitch G (2019). Does urbanization favour exotic bee species? Implications for the conservation of native bees in cities. Biol. Lett..

[36] Knapp S, Kühn I, Schweiger O, Klotz S (2008). Challenging urban species diversity: contrasting phylogenetic patterns across plant functional groups in Germany. Ecol. Lett..

[37] Kühn I, Brandl R, Klotz S (2004). The flora of German cities is naturally species rich. Evol. Ecol. Res..

[38] Knapp S, Winter M, Klotz S (2016). Increasing species richness but decreasing phylogenetic richness and divergence over a 320-year period of urbanization. J. Appl. Ecol..

[39] Lososová Z (2006). Patterns of plant traits in annual vegetation of man-made habitats in central Europe. Perspect. Plant Ecol. Evol. Syst..

[40] Pysek P (1998). Alien and native species in Central European urban floras: a quantitative comparison. J. Biogeogr..

[41] Schleuning M (2016). Ecological networks are more sensitive to plant than to animal extinction under climate change. Nat. Commun..

[42] Ollerton J (2017). Pollinator diversity: distribution, ecological function, and conservation. Annu. Rev. Ecol. Evol. Syst..

[43] Schleuning M, Fründ J, García D (2014). Predicting ecosystem functions from biodiversity and mutualistic networks: an extension of trait-based concepts to plant–animal interactions. Ecography.

[44] Mallinger RE, Gaines-Day HR, Gratton C (2017). Do managed bees have negative effects on wild bees?: A systematic review of the literature. PLoS ONE.

[45] Potts SG (2005). Role of nesting resources in organising diverse bee communities in a Mediterranean landscape. Ecol. Entomol..

[46] Pardee GL, Philpott SM (2014). Native plants are the bee’s knees: local and landscape predictors of bee richness and abundance in backyard gardens. Urban Ecosyst..

[47] Ballare KM, Neff JL, Ruppel R, Jha S (2019). Multi-scalar drivers of biodiversity: local management mediates wild bee community response to regional urbanization. Ecol. Appl..

[48] Torné-Noguera A (2014). Determinants of spatial distribution in a bee community: nesting resources, flower resources, and body size. PLoS ONE.

[49] Baldock KCR (2019). A systems approach reveals urban pollinator hotspots and conservation opportunities. Nat. Ecol. Evol..

[50] Fetridge ED, Ascher JS, Langellotto GA (2008). The bee fauna of residential gardens in a suburb of New York City (Hymenoptera: Apoidea). Ann. Entomol. Soc. Am..

[51] Stang M, Klinkhamer PGL, van der Meijden E (2006). Size constraints and flower abundance determine the number of interactions in a plant–flower visitor web. Oikos.

[52] Scolozzi R, Geneletti D (2012). A multi-scale qualitative approach to assess the impact of urbanization on natural habitats and their connectivity. Environ. Impact Assess. Rev..

[53] Cheptou P-O, Hargreaves AL, Bonte D, Jacquemyn H (2017). Adaptation to fragmentation: evolutionary dynamics driven by human influences. Philos. Trans. R. Soc. B Biol. Sci..

[54] Hennig EI, Ghazoul J (2011). Plant–pollinator interactions within the urban environment. Perspect. Plant Ecol. Evol. Syst..

[55] Winfree R, Aguilar R, Vázquez DP, LeBuhn G, Aizen MA (2009). A meta-analysis of bees’ responses to anthropogenic disturbance. Ecology.

[56] Quantum GIS Development Team. Quantum GIS Geographic Information System. Open Source Geospatial Foundation Project. Available at: http://qgis.osgeo.org. (2014).

[57] Greenleaf SS, Williams NM, Winfree R, Kremen C (2007). Bee foraging ranges and their relationship to body size. Oecologia.

[58] Westphal C (2008). Measuring bee diversity in different European habitats and biogeographical regions. Ecol. Monogr..

[59] Amiet, F. & Gesellschaft, S. E. *Insecta Helvetica. A, Fauna: 12. Hymenoptera. Apidae.-T. 1. Allgemeiner Teil, Gattungsschlüssel, Gattungen Apis, Bombus und Psithyrus*. (Musée d’Histoire naturelle, 1996).

[60] Amiet, F., Herrmann, M., Müller, A. & Neumeyer, R. Fauna Helvetica 6. Apidae 3: Halictus, Lasioglossum. *Fauna Helv. 6. Apidae 3 Halictus, Lasioglossum* (2001).

[61] Amiet, F., Müller, A. & Neumeyer, R. *Apidae 2: Colletes, Dufourea, Hylaeus, Nomia, Nomioides, Rhophitoides, Rophites, Sphecodes, Systropha*. **4** (Schweizerische Entomologische Gesellschaft, 1999).

[62] Hebert PDN, Cywinska A, Ball SL, de Waard JR (2003). Biological identifications through DNA barcodes. Proc. R. Soc. Lond. B Biol. Sci..

[63] Bäßler, M., Jäger, J. E. & Werner, K. *Rothmaler, W. (Begr.): Exkursionsflora von Deutschland. Bd.2: Gefäßpflanzen. 17.Aufl* (Berlin: Spektrum, 1999).

[64] Jäger, J. E., Wesche, K., Ritz, C., Müller, F. & Welk, E. *Rothmaler - Exkursionsflora von Deutschland, Gefäßpflanzen: Atlasband* (Springer-Verlag, 2013).

[65] Westrich, P. *Die Wildbienen Deutschlands* (Verlag Eugen Ulmer, 2018).

[66] Kattge J (2020). TRY plant trait database—enhanced coverage and open access. Glob. Change Biol..

[67] Botta-Dukát Z (2005). Rao’s quadratic entropy as a measure of functional diversity based on multiple traits. J. Veg. Sci..

[68] Laliberté E, Legendre P (2010). A distance-based framework for measuring functional diversity from multiple traits. Ecology.

[69] Rader R, Bartomeus I, Tylianakis JM, Lalibert E (2014). The winners and losers of land use intensification: pollinator community disassembly is non-random and alters functional diversity. Divers. Distrib..

[70] Faith DP (1992). Conservation evaluation and phylogenetic diversity. Biol. Conserv..

[71] Bartoń, K. MuMIn: Multi-Model Inference. R package version 1.15.1 (2013).

[72] Burnham KP, Anderson DR (2004). Multimodel inference. Sociol. Methods Res..

[73] Bates D, Mächler M, Bolker B, Walker S (2015). Fitting linear mixed-effects models using lme4. J. Stat. Softw..

[74] Legendre P, Galzin R, Harmelin-Vivien ML (1997). Relating behavior to habitat: solutions to the fourth-corner problem. Ecology.

[75] Wang, Y., Naumann, U., Eddelbuettel, D., Wilshire, J. & Warton, D. mvabund: Statistical Methods for Analysing Multivariate Abundance Data. R package version 4.1.3 (2020).

[76] Lefcheck JS (2016). piecewiseSEM: piecewise structural equation modelling in r for ecology, evolution, and systematics. Methods Ecol. Evol..

[77] Shipley B (2009). Confirmatory path analysis in a generalized multilevel context. Ecology.

[78] Sobel, M. E. Sociological methodology. In: *Sociological Methodology* (ed. Leinhart, S.) 290–312 (1982).

[79] Zuur A, Ieno EN, Walker N, Saveliev AA, Smith GM (2009). Mixed effects models and extensions in ecology with R.

[80] Paradis E, Claude J, Strimmer K (2004). APE: analyses of phylogenetics and evolution in R language. Bioinformatics.

[81] R Core Team. R: A Language and Environment for Statistical Computing. R Foundation for Statistical Computing. http://www.r-project.org (2016).

