# Supplementary material for: Urban fragmentation leads to lower floral diversity, with knock-on impacts on bee biodiversity
Source: Sci Rep. 2020 Dec 10;10:21756. doi: 10.1038/s41598-020-78736-x (PMC7730174; doi:10.1038/s41598-020-78736-x)
Supplement: Supplementary file 1 — Supplementary Information. [file 41598_2020_78736_MOESM1_ESM.docx]

**Supplementary Material**

**Urban fragmentation leads to lower floral diversity, with knock-on impacts on bee biodiversity**

Panagiotis Theodorou^1*^, Sarah-Christine Herbst^1^, Belinda Kahnt^1^, Patricia Landaverde-González^1,2^, Lucie M. Baltz^1^, Julia Osterman^1,3^, Robert J. Paxton^1,4^

^1^ General Zoology, Institute for Biology, Martin-Luther University Halle-Wittenberg, Hoher Weg 8, 06120 Halle (Saale), Germany

^2^ Unidad para el Conocimiento, Uso y Valoración de la Biodiversidad, Centro de Estudios Conservacionistas–CECON-, Facultad de Ciencias Químicas y Farmacia, Universidad de San Carlos de Guatemala, Avenida La Reforma 0-63 zona 10, 01010, Ciudad de Guatemala, Guatemala

^3^ Helmholtz Centre for Environmental Research-UFZ Leipzig, ESCALATE, Department of Computational Landscape Ecology, Permoserstrasse 15, 04318 Leipzig, Germany

^4^ German Centre for Integrative Biodiversity Research (iDiv) Halle-Jena-Leipzig, Deutscher Platz 5e, 04103 Leipzig, Germany

^*^ Correspondence:

Panagiotis Theodorou, General Zoology, Institute for Biology, Martin Luther University Halle-Wittenberg, Hoher Weg 8, 06120 Halle, Germany

Phone: +49 (0)345 55 26511, FAX: +49 (0)345 55 27428, Email: panatheod@gmail.com

**
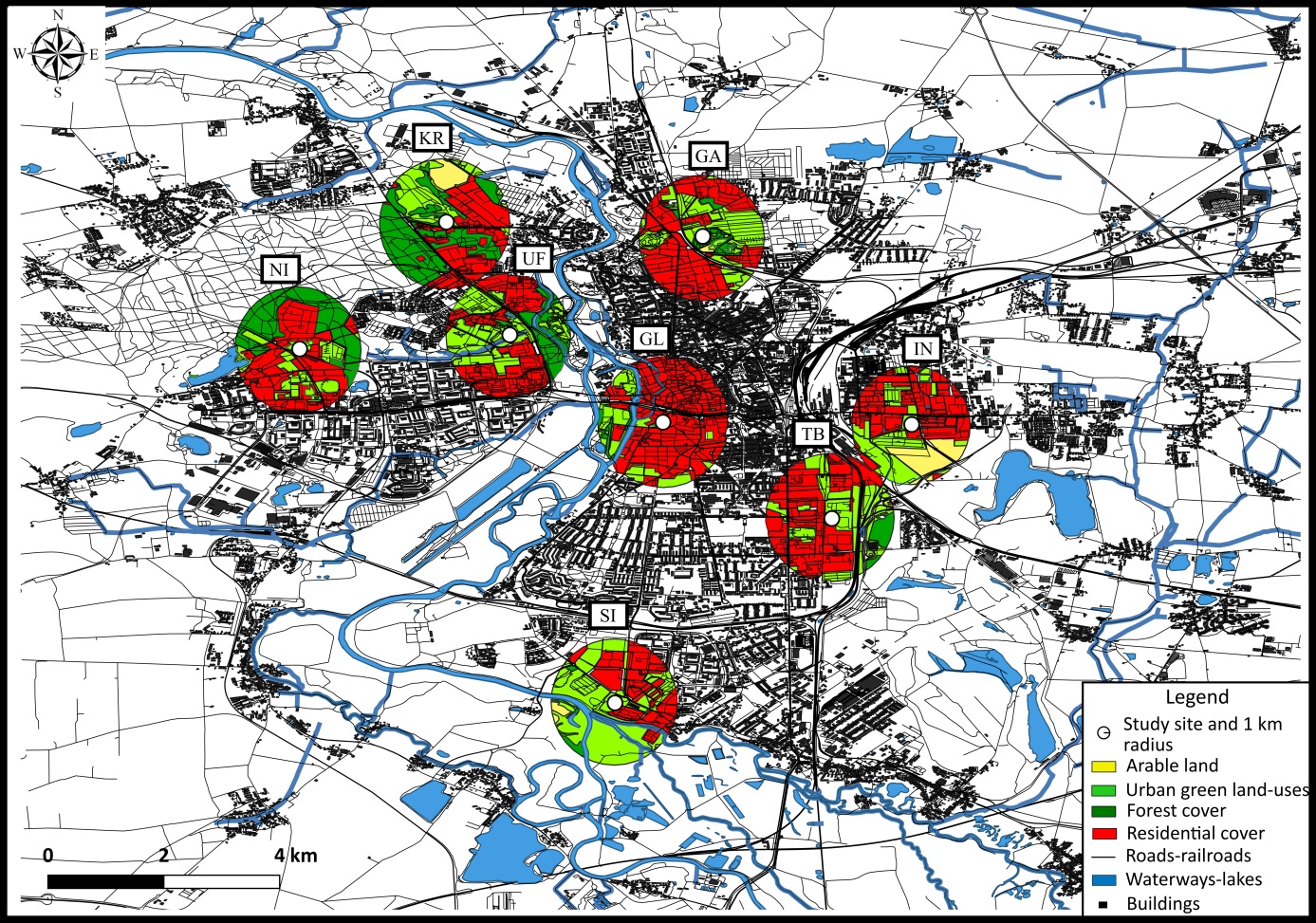
**

**Supplementary Figure 1.** The study area and eight study sites (2-letter codes; Supplementary Table S4) in the city of Halle (Saale), showing their landscape composition within a 1000 m radius of a site’s geographic centre. This map was created with OpenStreetMap vector data using Quantum GIS ^1^. This map is licensed under the Open Data Commons Open Database License (ODbL) by the OpenStreetMap Foundation (OSMF) (https://www.openstreetmap.org/copyright) (OpenStreetMap contributors).

**Table S1.** List of sampled bee species and their functional traits.

| Species | Average ITD (μm) | Sociality | Tongue | Nesting | Voltine | Lecty |
| --- | --- | --- | --- | --- | --- | --- |
| *Andrena alfkenella* | 1773.46 | Solitary | Short | Ground | Bivoltine | Polylectic |
| *Andrena florea* | 2793.96 | Solitary | Short | Ground | Univoltine | Oligolectic |
| *Andrena hypopolia* | 2465.50 | Solitary | Short | Ground | Bivoltine | Polylectic |
| *Andrena nitida* | 1983.05 | Solitary | Short | Ground | Bivoltine | Polylectic |
| *Andrena minutula* | 1256.19 | Solitary | Short | Ground | Bivoltine | Polylectic |
| *Andrena minutuloides* | 1276.53 | Solitary | Short | Ground | Bivoltine | Polylectic |
| *Andrena nitidiuscula* | 1212.30 | Solitary | Short | Ground | Univoltine | Oligolectic |
| *Andrena ovatula* | 2283.98 | Solitary | Short | Ground | Bivoltine | Polylectic |
| *Andrena wilkella* | 1963.58 | Solitary | Short | Ground | Univoltine | Oligolectic |
| *Anthophora aestivalis* | 3788.07 | Solitary | Long | Ground | Univoltine | Polylectic |
| *Anthophora quadrimaculata* | 3069.87 | Solitary | Long | Ground | Univoltine | Polylectic |
| *Bombus hortorum* | 4135.50 | Social | Long | Above Ground | Univoltine | Polylectic |
| *Bombus lapidarius* | 3600.76 | Social | Long | Ground | Univoltine | Polylectic |
| *Bombus lucorum* | 4119.88 | Social | Long | Ground | Univoltine | Polylectic |
| *Bombus pascuorum* | 3355.789 | Social | Long | Above Ground | Univoltine | Polylectic |
| *Bombus pratorum* | 3458.83 | Social | Long | Above Ground | Univoltine | Polylectic |
| *Bombus rupestris* | 3942.44 | Parasitic | Long | Ground | Univoltine |  |
| *Bombus sylvarum* | 3109.43 | Social | Long | Ground | Univoltine | Polylectic |
| *Bombus terrestris* | 3907.63 | Social | Long | Ground | Univoltine | Polylectic |
| *Bombus vestalis* | 4637.23 | Parasitic | Long | Ground | Univoltine |  |
| *Ceratina cyanea* | 1342.99 | Solitary | Long | Above ground | Univoltine | Polylectic |
| *Chelostoma campanularum* | 840.81 | Solitary | Long | Above ground | Univoltine | Oligolectic |
| *Coelioxys conica* | 2620.43 | Parasitic | Long | Ground | Univoltine |  |
| *Colletes daviesanus* | 2199.03 | Solitary | Short | Ground | Univoltine | Oligolectic |
| *Colletes fodiens* | 2474.87 | Solitary | Short | Ground | Univoltine | Oligolectic |
| *Dasypoda hirtipes* | 3004.57 | Solitary | Short | Ground | Univoltine | Oligolectic |
| *Epeolus variegatus* | 1953.98 | Parasitic | Long | Ground | Univoltine |  |
| *Eucera nigrescens* | 3365.41 | Solitary | Long | Ground | Univoltine | Oligolectic |
| *Halictus leucaheneus* | 1486.20 | Solitary | Short | Ground | Univoltine | Polylectic |
| *Halictus rubicundus* | 2440.77 | Social | Short | Ground | Bivoltine | Polylectic |
| *Halictus scabiosae* | 2415.91 | Solitary | Short | Ground | Univoltine | Polylectic |
| *Halictus simplex* | 1932.57 | Solitary | Short | Ground | Univoltine | Polylectic |
| *Halictus subauratus* | 1482.03 | Social | Short | Ground | Univoltine | Polylectic |
| *Halictus tumulorum* | 1458.91 | Social | Short | Ground | Univoltine | Polylectic |
| *Heriades truncorum* | 1359.97 | Solitary | Long | Above ground | Univoltine | Oligolectic |
| *Hylaeus brevicornis* | 938.36 | Solitary | Short | Above ground | Bivoltine | Polylectic |
| *Hylaeus dilatatus* | 1084.46 | Solitary | Short | Above ground | Univoltine | Polylectic |
| *Hylaeus nigritus* | 1291.16 | Solitary | Short | Above ground | Univoltine | Oligolectic |
| *Hylaeus punctatus* | 1217.44 | Solitary | Short | Above ground | Univoltine | Oligolectic |
| *Lasioglossum calceatum* | 1806.20 | Social | Short | Ground | Univoltine | Polylectic |
| *Lasioglossum laticeps* | 1400.62 | Social | Short | Ground | Univoltine | Polylectic |
| *Lasioglossum lativentre* | 1269.25 | Solitary | Short | Ground | Univoltine | Polylectic |
| *Lasioglossum leucozonium* | 1842.20 | Solitary | Short | Ground | Univoltine | Polylectic |
| *Lasioglossum malachurum* | 1523.74 | Social | Short | Ground | Univoltine | Polylectic |
| *Lasioglossum morio* | 1105.02 | Social | Short | Ground | Univoltine | Polylectic |
| *Lasioglossum pauxillum* | 1073.62 | Social | Short | Ground | Univoltine | Polylectic |
| *Lasioglossum politum* | 941.81 | Social | Short | Ground | Univoltine | Polylectic |
| *Lasioglossum villosulum* | 1289.44 | Solitary | Short | Ground | Bivoltine | Polylectic |
| *Macropis europaea* | 2209.80 | Solitary | Short | Ground | Univoltine | Oligolectic |
| *Megachile centuncularis* | 2543.86 | Solitary | Long | Above ground | Univoltine | Polylectic |
| *Megachile ericetorum* | 3144.94 | Solitary | Long | Above ground | Univoltine | Oligolectic |
| *Megachile maritima* | 3316.58 | Solitary | Long | Above ground | Univoltine | Polylectic |
| *Megachile rotundata* | 2294.89 | Solitary | Long | Above ground | Univoltine | Polylectic |
| *Megachile versicolor* | 2766.26 | Solitary | Long | Above ground | Bivoltine | Polylectic |
| *Megachile willughbiella* | 3365.15 | Solitary | Long | Above ground | Univoltine | Polylectic |
| *Nomada flavoguttata* | 1412.24 | Parasitic | Long | Ground | Bivoltine |  |
| *Osmia aurulenta* | 2809.00 | Solitary | Long | Above ground | Univoltine | Polylectic |
| *Osmia adunca* | 2751.03 | Solitary | Long | Above ground | Univoltine | Oligolectic |
| *Osmia anthocopoides* | 1418.45 | Solitary | Long | Above ground | Univoltine | Oligolectic |
| *Panurgus calcaratus* | 1695.41 | Solitary | Short | Ground | Univoltine | Oligolectic |
| *Pseudoanthidium nanum* | 1870.90 | Solitary | Long | Above ground | Univoltine | Oligolectic |
| *Sphecodes ferruginatus* | 1260.01 | Parasitic | Short | Ground | Univoltine |  |
| *Xylocopa violacea* | 5545.09 | Solitary | Long | Above ground | Univoltine | Polylectic |

**Table S2.** List of plant species and their functional traits. Breeding system, flower sex timing and plant longevity data were extracted from the TRY database v. 4.1 ^2–6^

| Species | Mean nectar holder depth (mm) | Flower shape | Flower color | Plant longevity | Breeding system | Flower sex timing |
| --- | --- | --- | --- | --- | --- | --- |
| *Achillea millefolium* | 1 | Open | White | Perennial | Allogamous | Protandrous |
| *Aegopodium podagraria* | 1 | Open | White | Perennial | Mixed mating | Protandrous |
| *Ballota nigra* | 14 | Papilionaceous | Violet | Perennial | Mixed mating | Protandrous |
| *Brassica napus* | 1 | Open | Yellow | Annual/biennial | Mixed mating | Protogynous |
| *Bryonia dioica* | 1 | Open | White | Perennial | Allogamous | Homogamous |
| *Calluna vulgaris* | 1 | Open | Violet | Perennial | Allogamous | Protandrous |
| *Centaurea diffusa* | 4 | Open | White | Annual/Biennial | Allogamous | Protandrous |
| *Centaurea jacea* | 4 | Open | Violet | Perennial | Allogamous | Protandrous |
| *Centaurea nigra* | 4 | Open | Violet | Perennial | Allogamous | Protandrous |
| *Centaurea scabiosa* | 4 | Open | Violet | Perennial | Allogamous | Protandrous |
| *Chamerion angustifolium* | 20 | Tubular | Violet | Perennial | Allogamous | Protandrous |
| *Cichorium intybus* | 1 | Open | Violet | Perennial | Allogamous | Protandrous |
| *Cirsium arvense* | 8 | Open | Red | Perennial | Allogamous | Protandrous |
| *Cirsium vulgare* | 8 | Open | Violet | Biennial | Mixed mating | Protandrous |
| *Clematis vitalba* | 1 | Open | White | Perennial | Autogamous | Protogynous |
| *Conyza canadensis* | 1 | Open | White | Annual/Biennial | Autogamous | Protandrous |
| *Coronilla varia* | 10 | Papilionaceous | White | Perennial | Allogamous | Homogamous |
| *Crepis biennis* | 1 | Open | Yellow | Biennial | Autogamous | Protandrous |
| *Daucus carota* | 1 | Open | White | Annual/Biennial | Allogamous | Protandrous |
| *Diplotaxis tenuifolia* | 1 | Open | Yellow | Perennial | Allogamous | Homogamous |
| *Echium vulgare* | 15.2 | Tubular | Violet | Biennial | Allogamous | Protandrous |
| *Epilobium hirsutum* | 27.4 | Tubular | Violet | Perennial | Allogamous | Protandrous |
| *Erigeron annuus* | 1 | Open | White | Annual/Biennial | Autogamous | Protandrous |
| *Galium mollugo* | 1 | Open | White | Perennial | Mixed mating | Homogamous |
| *Geum urbanum* | 1 | Open | Yellow | Perennial | Autogamous | Homogamous |
| *Hieracium vulgatum* | 1 | Open | Yellow | Perennial | Autogamous | Protandrous |
| *Hippocrepis comosa* | 13.5 | Papilionaceous | Yellow | Perennial | Allogamous | Homogamous |
| *Hypericum perforatum* | 1 | Open | Yellow | Perennial | Mixed mating | Homogamous |
| *Lamium purpureum* | 15 | Papilionaceous | Violet | Annual | Mixed mating | Homogamous |
| *Lathyrus pratensis* | 9.2 | Papilionaceous | Yellow | Perennial | Allogamous | Homogamous |
| *Lathyrus sylvestris* | 19.5 | Papilionaceous | Violet | Perennial | Allogamous | Homogamous |
| *Leontodon autumnalis* | 1 | Open | Yellow | Perennial | Allogamous | Protandrous |
| *Leucanthemum vulgare* | 3 | Open | White | Perennial | Mixedmaiting | Protandrous |
| *Linaria vulgaris* | 26.7 | Papilionaceous | Yellow | Perennial | Allogamous | Homogamous |
| *Lotus corniculatus* | 12 | Papilionaceous | Yellow | Perennial | Allogamous | Homogamous |
| *Matricaria chamomilla* | 3 | Open | White | Annual | Allogamous | Protandrous |
| *Medicago lupulina* | 2 | Papilionaceous | Yellow | Annual/Biennial | Autogamous | Homogamous |
| *Medicago sativa* | 13 | Papilionaceous | Violet | Perennial | Allogamous |  |
| *Melilotus alba* | 4.6 | Papilionaceous | White | Annual/Biennial | Autogamous | Homogamous |
| *Melilotus officinalis* | 6 | Papilionaceous | Yellow | Annual/Biennial | Allogamous | Homogamous |
| *Odontites verna* | 8.2 | Papilionaceous | Violet | Annual | Allogamous |  |
| *Onobrychis viciifolia* | 11.1 | Papilionaceous | Red | Perennial | Allogamous | Homogamous |
| *Papaver rhoeas* | 1 | Open | Red | Annual | Allogamous | Homogamous |
| *Picris echioides* | 1 | Open | Yellow | Annual/Biennial | Allogamous | Protandrous |
| *Potentilla reptans* | 1 | Open | Yellow | Perennial | Autogamous | Homogamous |
| *Rhinanthus minor* | 16.3 | Papilionaceous | Yellow | Annual | Mixed mating | Homogamous |
| *Rubus fruticosus* | 1 | Open | White | Perennial | Allogamous | Protogynous |
| *Senecio jacobaea* | 1 | Open | Yellow | Perennial | Allogamous | Protandrous |
| *Solidago canadensis* | 1 | Open | Yellow | Perennial | Allogamous | Protandrous |
| *Sonchus palustris* | 1 | Open | Yellow | Perennial | Allogamous | Protandrous |
| *Tanacetum vulgare* | 1 | Open | Yellow | Perennial |  | Protandrous |
| *Taraxacum officinale* | 1 | Open | Yellow | Perennial | Allogamous | Protandrous |
| *Thymus polytrichus* | 6.6 | Papilionaceous | Violet | Perennial | Allogamous | Protandrous |
| *Trifolium pratense* | 10.5 | Papilionaceous | Red | Perennial | Allogamous | Homogamous |
| *Trifolium repens* | 8 | Papilionaceous | White | Perennial | Allogamous | Homogamous |
| *Tripleurospermum inodorum* | 1 | Open | White | Annual/Biennial | Allogamous | Protandrous |
| *Verbascum thapsus* | 1 | Open | Yellow | Bienial | Mixed mating | Protogynous |
| *Vicia cracca* | 14.1 | Papilionaceous | Violet | Perennial | Allogamous | Homogamous |

**Table S3.** Spearman correlation coefficients (*rho*, below diagonal) for the relationships among bee species traits; significance of the relationship (adjusted *P* values using Holm’s method) is given above the diagonal, with significant values in bold. For nominal traits, trait values were coded as 0, 1 or 2. Life history: parasitic 0, solitary 1, social 2; Lecty: oligolectic 0, polylectic 1; Nesting: below-ground 0, above-ground 1; Tongue length: short 0, long 1; Voltinism: univoltine 0, bivoltine 1.

| Traits | ITD | Lecty | Nesting | Sociality | Tongue length | Voltinism |
| --- | --- | --- | --- | --- | --- | --- |
| ITD | - | 1 | 1 | 1 | **<0.001** | 1 |
| Lecty | 0.09 | - | 1 | **0.01** | 1 | 0.21 |
| Nesting | -0.06 | -0.19 | - | **0.01** | **0.001** | 1 |
| Sociality | 0.10 | 0.41 | -0.41 | - | 1 | 1 |
| Tongue length | 0.59 | 0.03 | 0.49 | 0.02 | - | 0.26 |
| Voltinism | -0.16 | 0.30 | -0.10 | -0.17 | -0.29 | - |

**Table S4.** Semi-natural patch size of sampling sites and the proportion of the main land cover classes at 1km radius.

| Site name | 2-letter code | Latitude | Longitude | Patch size (m^2^) | Proportion of allotment gardens | Proportion of semi-natural areas | Proportion of parks | Proportion of residential cover | Building cover (m^2^) |
| --- | --- | --- | --- | --- | --- | --- | --- | --- | --- |
| Galgenbergschlucht | GA | 51.50587 | 11.97167 | 718 | 0.266 | 0.021 | 0.025 | 0.586 | 270123 |
| Glauchaer Straße | GL | 51.47745 | 11.96444 | 4661 | 0.043 | 0.039 | 0.073 | 0.820 | 588904 |
| Industriegebiet | IN | 51.47633 | 12.00139 | 2315 | 0.180 | 0.006 | 0.006 | 0.587 | 227485 |
| Kröllwitz | KR | 51.50851 | 11.93083 | 56825 | 0.003 | 0.187 | 0.001 | 0.288 | 113343 |
| Nietleben | NI | 51.48882 | 11.90806 | 5158 | 0.055 | 0.050 | 0.013 | 0.479 | 163738 |
| Silberhöhe | SI | 51.43392 | 11.95694 | 34164 | 0.082 | 0.285 | 0.057 | 0.298 | 104694 |
| Thüringer Bahnhof | TB | 51.46252 | 11.99056 | 58848 | 0 | 0.230 | 0.048 | 0.569 | 294408 |
| UFZ | UF | 51.49437 | 11.94306 | 27742 | 0 | 0.133 | 0.220 | 0.286 | 218111 |

**Table S5.** Pearson correlation coefficients (r) of the relationship between bee species richness, abundance and flowering plant species richness with landscape diversity (measured as Shannon diversity of land-uses) at increasing area (given as radius in metres) from the centre of a site. The largest correlation coefficient is given in bold.

| Radius | 250 m | 500 m | 750 m | 1,000 m | 1,250 m | 1,500 m |
| --- | --- | --- | --- | --- | --- | --- |
| Bee richness | -0.20 | -0.12 | -0.15 | **-0.25** | 0.12 | 0.04 |
| Bee abundance | 0.08 | 0.13 | 0.22 | **0.37** | 0.34 | 0.31 |
| Plant richness | -0.10 | -0.04 | 0.17 | **0.29** | 0.28 | 0.25 |

**Supplementary Methods 1**

The species identity of 209 bees that could not be identified unambiguously by morphological traits was confirmed by DNA barcoding. To do so we extracted genomic DNA from one or two mid-legs (depending on bee size) using a Chelex extraction protocol ^7^. The ca. 650 bp region of the mitochondrial cytochrome *c* oxidase subunit I (COI) gene was then amplified using DNA extracts with universal primers LCO-1490 (5'-GGTCAACAAATCATAAAGATATTGG-3') and HCO-2198 (5'-TAAACTTCAGGGTGACCAAAAAATCA-3') ^8^. PCR reactions were carried out in 10 µL volumes consisting of 1 x PCR buffer containing 1.5 mM MgCl_2_ (Promega, Madison, WI, USA), 200 µM of each dNTP, 0.4 µM of each primer, 1.5 U *Taq*-Polymerase (Promega) and 2 µL of template DNA (ca. 25-50 ng). PCRs were performed with a Biometra TProfessional basic gradient thermocycler (Biometra, Göttingen, Germany) under the following thermal regime: 3 min at 94°C, followed by 36 cycles of 30s at 94°C, 45s annealing at 50°C and 1 min at 72°C for elongation and a final elongation step at 72°C for 8 min. PCR products were screened with a QiAxcel capillary electrophoresis system (Qiagen, Hilden, Germany) to confirm a single PCR product of the correct size had been amplified. PCR products were purified using an ExoSAP-IT PCR Product Cleanup kit (Affymetrix, Santa Clara, CA, USA; Bell, 2008) and sequenced commercially on an ABI 3730xl DNA autosequencer with the LCO-1490 primer.

The resulting chromatograms were checked by eye, sequences were trimmed, manually aligned and checked for potential open reading frame shifts and premature stop codons in Geneious v.7.1.9 ([https://www.geneious.com](http://www.geneious.com/)). Sequences were BLASTed against the NCBI GenBank nucleotide and the Barcode of Life (BOLD) databases ^9^. We used a threshold BLAST hit similarity higher than 98 % for species identification.

**Supplementary Methods 2**

To estimate phylogenetic diversity we build bee and plant phylogenies based on two genes for each taxon (bee: COI and elongation factor 1-alpha [ef1-alpha]; plant: ribulose-1,5-bisphosphate carboxylase/oxygenase large subunit [rbcL] and maturase K [matK]), which were downloaded from NCBI GenBank. We aligned sequences of each gene separately using the Geneious algorithm implemented in Geneious v. 7.0.6. ([https://www.geneious.com](http://www.geneious.com/)), manually edited the alignments and excluded highly ambiguous aligned positions. Due to several indels, we first aligned ef1-alpha sequences with MUSCLE ^10^ and thereafter re-aligned them in Geneious using the Geneious algorithm and manual editing. Alignments of individual bee or plant genes were then concatenated using SequenceMatrix v. 1.8 ^11^ (alignment length plant = 936 bp, bee = 1,664 bp). We inferred the best fitting evolutionary model based on model comparisons via the Akaike criterion using MrModeltest v. 2.3 ^12^ and PAUP v. 4.0a ^13^. Phylogenetic reconstructions were then carried out in MrBayes v. 3.2.6 ^14^ at the CIPRES Science Gateway v. 3.1 ^15^ using the most suitable evolutionary model found with MrModeltest (COI= SYM+G, ef1-alpha= SYM+G, rbcL= K2+G+I, matK= GTR+G+I). Both datasets, bee and plant alignment, were analysed in MrBayes; we performed two runs, each with four parallel MCMC chains with 1,000,000 generations, sampling every 100 generations. Convergence of the MCMC chains was confirmed in Tracer v. 1.5 ^16^. We discarded the first 2,500 trees of each run (burn-in) and the remaining tree samples from both runs were combined to infer a consensus tree.

**References**

1. Quantum GIS Development Team. Quantum GIS Geographic Information System. *Open Source Geospatial Foundation Project* (2015).

2. Kattge, J. *et al.* TRY plant trait database – enhanced coverage and open access. *Glob. Chang. Biol.* **26**, 119–188 (2020).

3. Kühn, I., Durka, W. & Klotz, S. BiolFlor - A new plant-trait database as a tool for plant invasion ecology. *Diversity and Distributions* **10**, 363-365 (2004).

4. Moretti, M. & Legg, C. Combining plant and animal traits to assess community functional responses to disturbance. *Ecography* **32**, 299–309 (2009).

5. Bragazza, L. Conservation priority of Italian Alpine habitats: a floristic approach based on potential distribution of vascular plant species. *Biodivers. Conserv.* **18**, 2823–2835 (2009).

6. Wirth, C. & Lichstein, J. W. The Imprint of Species Turnover on Old-Growth Forest Carbon Balances - Insights From a Trait-Based Model of Forest Dynamics. In: Wirth C., Gleixner G., Heimann M. (eds) Old-Growth Forests. Ecological Studies (Analysis and Synthesis), vol 207. Springer, Berlin, Heidelberg (2009).

7. Walsh, P. S., Metzger, D. A. & Higuchi, R. Chelex 100 as a medium for simple extraction of DNA for PCR-based typing from forensic material. *Biotechniques* **10**, 506–13 (1991).

8. Folmer, O., Black, M., Hoeh, W., Lutz, R. & Vrijenhoek, R. DNA primers for amplification of mitochondrial cytochrome c oxidase subunit I from diverse metazoan invertebrates. *Mol. Mar. Biol. Biotechnol.* **3**, 294–9 (1994).

9. Ratnasingham, S. & Hebert, P. D. N. bold: The Barcode of Life Data System (http://www.barcodinglife.org). *Mol. Ecol. Notes* **7**, 355–364 (2007).

10. Edgar, R. C. MUSCLE: multiple sequence alignment with high accuracy and high throughput. *Nucleic Acids Res.* **32**, 1792–7 (2004).

11. Vaidya, G., Lohman, D. J. & Meier, R. SequenceMatrix: concatenation software for the fast assembly of multi-gene datasets with character set and codon information. *Cladistics* **27**, 171–180 (2011).

12. Nylander, J. A. A. MrModeltest v2. *Evolutionary Biology Centre, Uppsala University* (2004).

13. Swofford, D. L. PAUP*: phylogenetic analysis using parsimony, version 4.0b10. *21 Libr.* (2003).

14. Huelsenbeck, J. P. & Ronquist, F. MRBAYES: Bayesian inference of phylogenetic trees. *Bioinformatics* **17**, 754–5 (2001).

15. Miller, M. A., Pfeiffer, W. & Schwartz, T. Creating the CIPRES Science Gateway for inference of large phylogenetic trees. in *2010 Gateway Computing Environments Workshop, GCE 2010* (2010).

16. Rambaut, A., Drummond, A. J. & Rambaut, A. Tracer V1.5. *Available from http//beast.bio.ed.ac.uk/Tracer* (2009).
